# Editorial: Rhizosphere Spatiotemporal Organisation

**DOI:** 10.3389/fpls.2021.795136

**Published:** 2021-11-18

**Authors:** Mika T. Tarkka, Michael Bonkowski, Tida Ge, Claudia Knief, Bahar S. Razavi, Doris Vetterlein

**Affiliations:** ^1^Department of Soil Ecology, Helmholtz Centre for Environmental Research – UFZ, Halle, Germany; ^2^German Centre for Integrative Biodiversity Research (iDiv) Halle-Jena-Leipzig, Leipzig, Germany; ^3^Terrestrial Ecology, Institute of Zoology, University of Cologne, Cologne, Germany; ^4^Key Laboratory of Agroecological Processes in Subtropical Region, Institute of Subtropical Agriculture, Chinese Academy of Sciences, Changsha, China; ^5^Molecular Biology of the Rhizosphere, Crop Functional Genomics, Institute of Crop Science and Resource Conservation (INRES), University of Bonn, Bonn, Germany; ^6^Department of Soil and Plant Microbiome, Institute of Phytopathology, Christian- Albrechts-University of Kiel, Kiel, Germany; ^7^Department of Soil System Science, Helmholtz Centre for Environmental Research – UFZ, Halle, Germany

**Keywords:** soil properties, modeling, biosensor, network analysis, community assembly, process rate

Formation of the rhizosphere, interface between living plant roots and soil, leads to changes in soil properties, nutrient and water distribution and biogeochemical cycling, and to a selection of unique populations of microorganisms and invertebrates. Dynamic feedback processes between the plant, the soil and the biota govern rhizosphere formation. The Frontiers Research Topic on “*Rhizosphere Spatiotemporal Organization*” presents contributions which aim to advance our understanding of rhizosphere processes. All of the six articles took the challenge to elaborate on the dynamic interactions and feedback processes in both spatial and temporal contexts.

The dynamics of water uptake by roots in soil is influenced by both soil bulk density and rhizodeposition. Rhizosphere soil can be less porous than bulk soil due to increased aggregation and compression (Daly et al., 2015), but the immediate root-soil interface is a zone of high porosity (Helliwell et al., 2019). Landl et al. investigated how maize roots influence both rhizosphere hydraulic properties and bulk density. They identified gradients in bulk density and mucilage concentrations based on X-ray scans and used a mechanistic simulation model to evaluate the impact of these gradients on soil water dynamics. According to the simulation results, the modification of rhizosphere bulk density and mucilage concentration by plant roots appear as a means to reduce water stress and to reduce root water uptake under drought. The authors suggest that the changes in rhizosphere bulk density and mucilage viscosity keep transpiration at a lower level for a longer time and may thus prevent dehydration.

Ethylene is a plant hormone that plays fundamental roles in root branching, root hair formation, and plant response to stress. The ethylene precursor 1-aminocyclo-propane-1-carboxylate (ACC) is scavenged by a specific group of plant growth promoting rhizobacteria possessing the enzyme ACC deaminase, encoded by the *acdS* gene (Bouffaud et al., 2018). By the action of this enzyme, the bacteria lower the levels of ethylene in roots and promote plant stress tolerance (Glick, 2014). By analyzing the rhizosphere communities of 3-week-old maize, Gebauer et al. showed that the populations of *acdS* carrying microorganisms in the rhizosphere are strongly affected by soil properties and rooting depth in soil columns, but only little by the presence of root hairs. These findings suggest that soil environmental constraints (i.e., environmental filters for community selection) may play a much stronger role than root hairs in the assembly of *acdS* carrying microbes on plant roots, and suggest that different soils and rooting depths sustain the diversity of *acdS* carrying microorganisms.

Rhizosphere metabolite concentration gradients serve as cues for rhizosphere organisms, including plant pathogens (Bouwmeester et al., 2003; Baker et al., 2005). Virulent *Agrobacterium tumefaciens* detects wounded living roots in soil by a gated receptor that requires simultaneous perception of two plant metabolites, exuded sugars and phenolics to induce virulence gene expression. By constructing separate biosensor strains for sugars and phenolics of *A. tumefaciens*, Liu et al. were able to correlate metabolite concentrations with the behavior of this bacterial pathogen. In this way, the spatiotemporal distribution of acetosyringone, a flavonoid that contributes to plant infection (Baker et al., 2005), and sugars, was measured. Wound-induced acetosyringone peaks accumulated in *A. tumefaciens* infected dicot (tobacco), but not monocot (maize). By contrast, both tobacco and maize wound sites contain sufficient sugar to induce the sugar responsive strain. The data thus support the view of fundamental specific importance of acetosyringone in plant tissue colonization by *A. tumefaciens*.

Since the rhizosphere is a dynamic system of feedback operating among interacting physical, chemical, and biological processes, it has been argued that a holistic rhizosphere view is needed, integrating these components (York et al., 2016). One approach for this is to apply the concept of self-organization, where order is created by interactions of processes amongst themselves rather than through external intervention. The review of Vetterlein et al. argues that the resilience of the rhizosphere, its ability to maintain its state during environmental stress, or to recover its functions after destabilizing influences, emerges from self-organized spatiotemporal patterns. The authors also suggest how the emergent system properties, such as water and nutrient availability or plant health, can be reached by multidisciplinary rhizosphere research.

In the contexts of space and time, important questions are when and where a predictable rhizosphere microbiome is formed along the root axis and by which factors the stable formation of a microbiome is challenged. Choosing maize as a rhizosphere model species, Bonkowski et al. tackle these questions in their mini-review. Plant impacts altered by root zones and types, developmental stages, circadian rhythm, and patterns of rhizodeposition are first treated in respect to microbiome assembly. An interesting part considers microbiome assembly from a microbial perspective, which is based on the concept of community assembly (Kraft et al., 2007; Thrall et al., 2007; Cavender-Bares et al., 2009). Since understanding of the interactions between bacteria, fungi and their grazers, protists, has greatly advanced during the past years of intensive research (Koller et al., 2013; Amacker et al., 2020; Rozmoš et al., 2021), we can now appreciate the central role of predator-prey dynamics on the assembly of the rhizosphere microbiome. The authors point at the central role of omics techniques and network analyses in the future studies.

With a focus on microbial processes and traits in a spatiotemporal context in the rhizosphere the review of Blagodatskaya et al. links the rhizosphere with interacting soil spheres: the detritussphere, created by dead material (Marschner et al., 2012), the porosphere, especially biopores created by plant roots or earthworms (Banfield et al., 2018), and surfaces of soil aggregates (Wilpiszeski et al., 2019). The key suggestion of the authors is that healthy soil emerges from specialized microbial communities and trophic interactions among them. The authors emphasize the central rule of the microorganisms as the main players in soil interfaces, and argue that the microorganisms use functional traits such as production of specific extracellular enzymes as a tool to develop a life strategy. They then show how resulting functional traits within and between these interfaces and the transformation of organic material lead to distinct process rates. As a perspective, further work on precise localization of biochemical processes within and between the interfaces is suggested, by appreciating the interactions among the microbes and the trophic chain, and environmental constraints. Of note, an approach underlined by the Bonkowski et al. minireview as well.

The research articles and reviews contributing to the Research Topic “*Rhizosphere Spatiotemporal Organization*” revealed interesting and complementary details, and led us to realize how a holistic view—overreaching different disciplines of rhizosphere research and taking both time and space in concern—is central for deeper understanding of this plant driven dynamic hotspot in soil (Cardon and Whitbeck, 2007; Hinsinger et al., 2009; Kuzyakov and Blagodatskaya, 2015). With this Research Topic, we hope to present a stimulus for continuing discussion in this rapidly developing field, and feel that our understanding of the complexity of rhizosphere processes is on a good way.

## Author Contributions

All authors listed have made a substantial, direct and intellectual contribution to the work, and approved it for publication.

## Conflict of Interest

The authors declare that the research was conducted in the absence of any commercial or financial relationships that could be construed as a potential conflict of interest.

## Publisher's Note

All claims expressed in this article are solely those of the authors and do not necessarily represent those of their affiliated organizations, or those of the publisher, the editors and the reviewers. Any product that may be evaluated in this article, or claim that may be made by its manufacturer, is not guaranteed or endorsed by the publisher.
